# Sleeping More Hours Per Day Than Working Can Prevent New-Onset Diabetes

**DOI:** 10.3389/ijph.2023.1606634

**Published:** 2023-12-08

**Authors:** Haiyan Yu, Xiaoyu Zhao, Xiaodong Zhang, Haishan Wei, Anju Zuo, Yuan Guo

**Affiliations:** ^1^ Department of General Practice, Qilu Hospital of Shandong University, Jinan, Shandong, China; ^2^ Central Hospital Affiliated to Shandong First Medical University, Jinan, Shandong, China

**Keywords:** sleep duration, work hours, sleep hours/work hours ratio, new-onset diabetes, prevention

## Abstract

**Objectives:** We expressed the combined effect by the ratio of daily sleep time to daily work time. The aim of this study was to discussed the predictive ability of daily sleep hours/work hours (SH/WH) ratio for diabetes risk.

**Methods:** Cox proportional hazards regression was used to calculate the hazard ratios (HRs) of new-onset diabetes. Restricted cubic spline analyses were performed to visualize the influence trend of SH/WH ratio and diabetes risk.

**Results:** The RCS model revealed a non-linear and L-shaped correlation between SH/WH ratio and diabetes risk. Compared with the participates with SH/WH ratio <1, those with a ratio ≥1 had a lower risk of developing diabetes. The multivariable adjusted hazard ratios (HRs) with 95% confidence intervals (CIs) of new-onset diabetes in Q2, Q3, Q4 and Q5 groups compared with Q1 group were 0.82 (0.57, 1.19), 1.05 (0.69, 1.59), 0.57 (0.36, 0.91), 0.66 (0.42, 1.06). The Kaplan-Meier curve showed that Q4 group had lower cumulative incidence.

**Conclusion:** Sleeping longer than working (SH/WH ratio ≥1) can reduce risk for developing diabetes. A minimal risk observed at 1.10–<1.37 (the fourth quintile) of SH/WH ratio.

## Introduction

Diabetes mellitus is a serious public health problem worldwide [1]. Global diabetes-related health expenditures were estimated at 966 billion USD in 2021, and are projected to reach 1,054 billion USD by 2045 [2]. Of the estimated 463 million adults with diabetes mellitus globally nearly half live in two large countries: India and China [3, 4]. With the aging population, economic development, urbanization, unhealthy dietary habits and sedentary lifestyle, diabetes mellitus has become a common disease in China. Diabetes and its complications are important causes of the burden of death and disability in China [5]. Therefore, screening for predictors and high-risk populations of diabetes is particularly important for early prevention of diabetes.

It has been demonstrated that sleep duration was closely related to the occurrence of diabetes. Some studies have found that both short and long sleep duration are associated with the risk of diabetes [6–8]. However, the conclusions on the optimal sleep duration are inconsistent. It has been suggested that the lowest diabetes risk is 7–8 h of sleep per day [6]. A reaction study in China have found that the best sleep time is between 6.3 and 7.5 h per night to minimize the risk of developing diabetes [9]. Although there are few studies on working hours and diabetes, the current results support that long working hours significantly increase the risk of diabetes [10–12].

Notably, there is still a lack of studies that consider both sleep and work duration to predict the occurrence and progression of diabetes in the Chinese population. We expressed the combined effect by the ratio of daily sleep time to daily work time. Therefore, based on data from the China Health and Nutrition Survey (CHNS), the aim of this study was to investigate the association of sleep hours/work hours (SH/WH) ratio with risk of diabetes in the general adults.

## Methods

### Study Subjects

**FIGURE 1:**
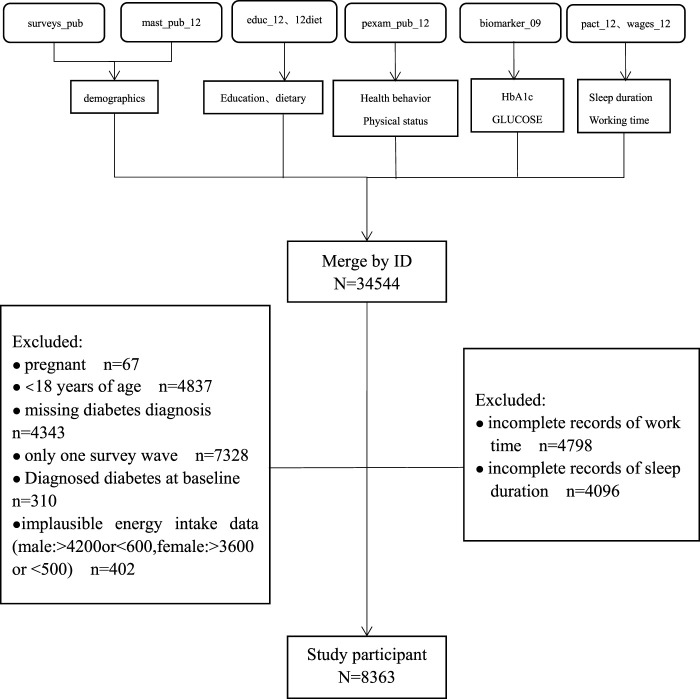
Flow chart for screening subjects (China, 2022).

The CHNS is an ongoing, open, longitudinal cohort survey that began in 1989 and has completed 10 rounds of surveys (1989, 1991, 1993, 1997, 2000, 2004, 2006, 2009, 2011, and 2015). Details of the study design and some findings of the CHNS have been described elsewhere [13–15]. The original data for this study was obtained from a publicly available dataset within the China Health and Nutrition Examination Survey (CHNS) 2004–2015. The initial study cohort included a total of 34,544 participants in China. Participants who met the following criteria had been excluded: < 18 years of age (*n* = 4837), only one survey wave (*n* = 7328), baseline diagnosis of diabetes (*n* = 310), missing diabetes diagnosis (*n* = 4,343), pregnant (*n* = 67), no available work time (*n* = 4,798), no available sleep duration (*n* = 4,096), extreme dietary energy data (male: >4,200 kcal or <600 kcal, female >3,600 kcal or <500 kcal) (*n* = 402). Finally, a total of 8,363 participants were recruited into our study cohort (Figure 1).

This survey was approved by the institutional review boards of the University of North Carolina at Chapel Hill and the National Institute of Nutrition and Food Safety, and the Chinese Center for Disease Control and Prevention. Each participant voluntarily signed a written informed consent before recruitment. All methods were performed in accordance with the relevant guidelines and regulations. The data and study materials that support the findings of this study can be found from the CHNS official website (http://www.cpc.unc.edu/projects/china).

### Sleep Hours/Work Hours (SH/WH) Ratio

Sleep duration was derived from self-report questionnaires with a question “How many hours each day do you usually sleep, including during both daytime and nighttime?”.

Working time was derived from self-report questionnaires with a question “For how many hours in a day, on the average, did you work?”.

In this study, the cumulative average length of sleep and work for each participant during the last round visit period from baseline to the date of new onset of diabetes or the end of follow-up was calculated to represent for long-term sleep and work duration and minimize within-person variation.

### Covariates Measurements

Lifestyle and demographic information were collected through questionnaires, including sex, age, urban or rural residents, education levels and smoking, alcohol, tea and coffee consumption status. Body mass index (BMI) was a ratio of weight (kg) in comparison to height squared (m^2^). After the participants rested for 5 min, blood pressure was measured in a standard way by trained researchers using a mercury manometer. Mean systolic and mean diastolic blood pressure from three independent measurements in the same arm were used.

### Outcome

Through the interview in the questionnaire survey: “Has a doctor ever told you that you suffer from diabetes?” Participants who answered “yes” were defined as having new-onset diabetes.

The year in which participants were first included in the survey was considered as the baseline. Person-years of follow-up for each participant were calculated from baseline until the first diagnosis with diabetes (the middle date between the survey of the first diagnosis and the nearest survey before), the last survey round before the participant was lost to follow-up, or until being censored at the end of the follow-up period, whichever came first. Incidence rates of diabetes, expressed as per 1,000 person years, were calculated as the number of new-onset diabetes cases divided by the person-years of follow-up.

### Statistical Analysis

The ANOVA test was used for normally distributed variables, while the Wilcoxon rank-sum test was used for non-normally distributed variables. All continuous variables are expressed as mean (SD) or medians (interquartile ranges). Differences in categorical variables were examined by the chi-square test and shown as number (n) and percentage (%).

We first divided participants into two groups: (SH/WH) ratio <1 (those who slept shorter than working hours) and SH/WH ratio ≥1 (those who slept equal to or longer than working hours). Cox proportional hazards regression was used to estimate the association of SH/WH ratio and new-onset diabetes. Furthermore, all participants were divided equally into five groups according to the quintiles of SH/WH ratio, including Q1 group (<0.89), Q2 group (0.89–<1.00), Q3 group (1.00–<1.10), Q4 group (1.10–<1.37) and Q5 group (≥1.37). Then, Kaplan-Meier curves were performed to describe the cumulative incidence of diabetes and differences between groups were estimated with the log-rank test. In addition, we performed restricted cubic spline (RCS) Cox regression, with 4 knots (20th, 40th, 60th, 80th percentiles of SH/WH), to evaluate the shape of dose–response association of SH/WH ratio and the risk of diabetes.

R software, version 4.2.3 and STATA 17.0 was used for all data analyses. A *p-*value < 0.05 (two-sided) was considered statistically significant.

## Results

Table 1 shows the baseline characteristics of the researchers. Compared to participants with SH/WH ratio <1, participants with SH/WH ratio ≥1 were older and had a higher proportion of females. In addition, participants with SH/WH ratio ≥1 were more likely to live in rural areas, had lower levels of education, had lower BMI, systolic blood pressure, diastolic blood pressure, fat intake, protein intake and working time, higher energy intake, carbohydrate intake and sleep duration. Notably, the proportions of smoking, drinking alcohol, drinking tea and coffee were lower than those in the group of (SH/WH) ratio <1 (all *p* < 0.05).

**TABLE 1:** Population characteristics by sleep hours/work hours ratio (<1 and ≥1) (China, 2022).

Factor	(SH/WH) ratio <1	(SH/WH) ratio ≥1	*p*-value
N	3,077	5,286	
Age, year	42.3 (12.3)	44.0 (13.2)	<0.01
Male (%)	1731 (56.3)	2,609 (49.4)	<0.01
Education level (%)			<0.01
Illiteracy	258 (8.4)	880 (16.6)	
Primary school	471 (15.3)	1,209 (22.9)	
Middle school	1,117 (36.3)	1724 (32.6)	
High school or above	1,231 (40.0)	1,473 (27.9)	
Urban (%)	1,449 (47.1)	1,401 (26.5)	<0.01
BMI, (kg/m^2^)	23.5 (3.3)	23.1 (3.3)	<0.01
SBP, mmHg	120.8 (16.1)	120.3 (17.1)	0.16
DBP, mmHg	78.8 (10.5)	78.2 (10.9)	0.02
Smoking (%)	1,145 (37.2)	1732 (32.8)	<0.01
Drinking alcohol (%)	1,223 (40.1)	1834 (35.0)	<0.01
Drinking tea (%)	1,324 (43.1)	1958 (37.1)	<0.01
Drinking coffee (%)	201 (6.5)	173 (3.3)	<0.01
Dietary intake			
Energy, Kcal/day	2046.5 (1642.0, 2518.6)	2126.6 (1708.5, 2573.5)	<0.01
Carbohydrate, g/day	271.4 (209.1, 348.8)	307.7 (236.6, 385.0)	<0.01
Fat, g/day	70.6 (47.2, 93.8)	63.3 (41.9, 88.1)	<0.01
Protein, g/day	65.3 (51.0, 81.1)	63.9 (51.0, 79.7)	0.04
Sleep duration, hours/day	7.5 (0.7)	8.2 (0.7)	<0.01
Working time, hours/day	8.7 (1.2)	6.5 (1.6)	<0.01

Abbreviations: BMI, body mass index; SBP, systolic blood pressure; DBP, diastolic blood pressure; HR, hazard ratio; CI, confidence interval.

**FIGURE 2:**
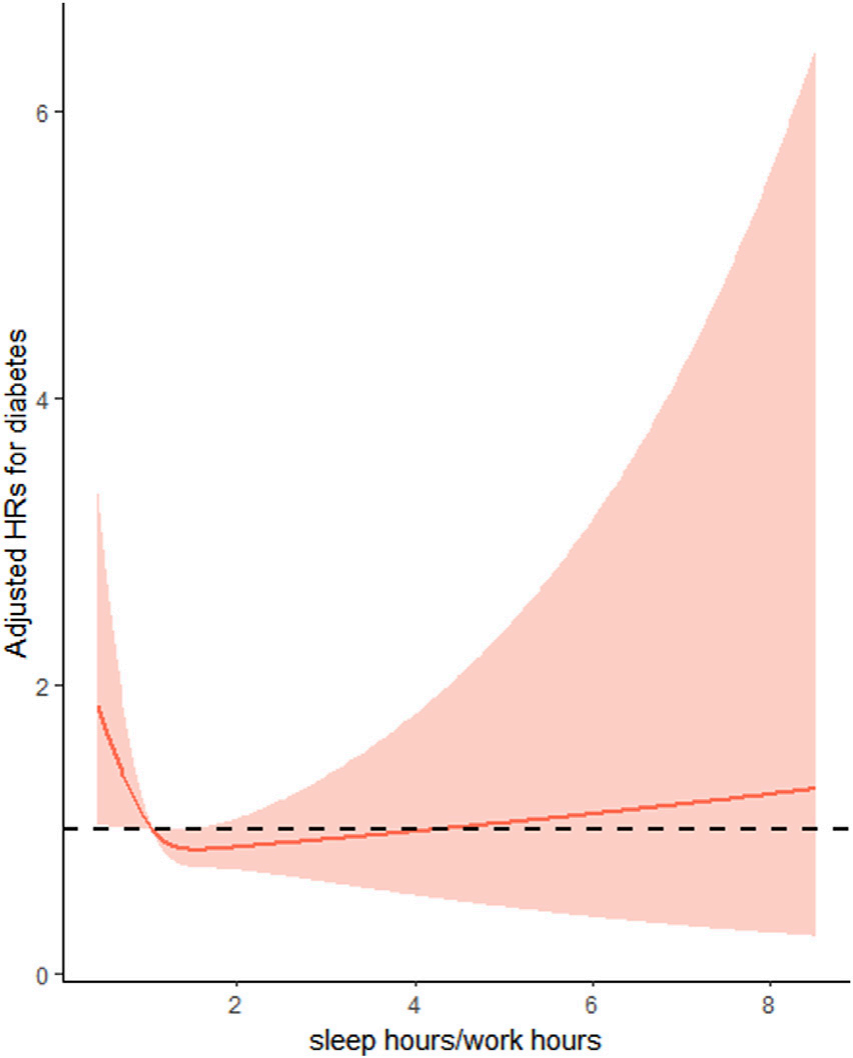
Restricted cubic spline Cox regression of the association of sleep hours/work hours ratio and the risk of new-onset diabetes (China, 2022). Adjusted for age at baseline (<45, 45-<60, ≥60), sex (male/female), residence (urban or rural) and education (Illiteracy, Primary school, middle school, high school or above) BMI (<24 kg/m^2^, ≥24 kg/m^2^), SBP (<140 mmHg, ≥140 mmHg), DBP (<90 mmHg, ≥90 mmHg), smoking status (yes/no), alcohol consumption (yes/no), drinking tea (yes/no), drinking coffee (yes/no), total energy intake (continuous), total fat intake (continuous), total carbohydrate intake (continuous) and total protein intake (continuous).

The final cohort analysis included 8,363 participants of whom 232 (4.10 per 1,000 person-years) developed diabetes during a median follow-up period of 7.0 years. Overall, the association between SH/WH ratio with the risk of new-onset diabetes followed a L-shape (*P* for non-linearity <0.001) (Figure 2).

Consistently, when SH/WH ratio divided into two groups: SH/WH ratio <1 and SH/WH ratio ≥1, compared with participants with SH/WH ratio <1 (5.3 per 1,000 person-years), the incidence of diabetes was significantly lower in those with (SH/WH) ratio ≥1 (3.5 per 1,000 person-years). In the initial unadjusted model, (SH/WH) ratio ≥1 was shown to reduce the risk of diabetes (HR: 0.66, 95% CI: 0.51–0.86, *p* < 0.001) compared with (SH/WH) ratio <1. After adjusting for potential variables such as age, sex, education level, BMI and alcohol consumption, the result remained significant, with an HR of 0.75 (95% CI: 0.57–0.99) (Table 2).

**TABLE 2:** The association between sleep hours/work hours ratio (<1 and ≥1) and the risk of new-onset diabetes (China, 2022).

Sleep hours/work hours (everyday)	No. of case (incidence rate)	Person years	Model 1	Model 2	Model 3
			HR (95% CI)	HR (95% CI)	HR (95% CI)
<1	106 (5.3)	20,092	Ref	Ref	Ref
≥1	126 (3.5)	36,496	0.66 (0.51–0.86) **	0.69 (0.53–0.91) **	0.75 (0.57–0.99) *

*p < 0.05, **p < 0.01, ***p < 0.001.

Model 2: adjusted for age at baseline (<45, 45-<60, ≥60), sex (male/female), residence (urban or rural) and education (Illiteracy, Primary school, middle school, high school or above).

Model 3 was further adjusted for BMI (<24 kg/m^2^, ≥24 kg/m^2^), SBP (<140 mmHg, ≥140 mmHg), DBP (<90 mmHg, ≥90 mmHg), smoking status (yes/no), alcohol consumption (yes/no), drinking tea (yes/no), drinking coffee (yes/no), total energy intake (continuous), total fat intake (continuous), total carbohydrate intake (continuous) and total protein intake (continuous).

Abbreviations: BMI, body mass index; SBP, systolic blood pressure; DBP, diastolic blood pressure; HR, hazard ratio; CI, confidence interval.

Next, SH/WH ratio was assessed as quintiles, with the first quintile (<0.89) as a baseline, the adjusted hazard ratios (HRs) and 95% confidence intervals (CIs) of diabetes were 0.82 (0.57, 1.19), 1.05 (0.69, 1.59), 0.57 (0.36, 0.91) and 0.66 (0.42, 1.06), respectively, for the second quintile (0.89 -<1.00), third quintile (1.00 -<1.10), fourth quintile (1.10 -<1.37), and fifth quintile (≥1.37) after adjustments for age, region, education level, BMI, SBP, DBP, smoking status, drinking status, drinking tea, drinking coffee, and dietary intake (Energy, Carbohydrate, Fat, Protein) (Table 3). The lower risk of new-onset diabetes was found in subjects in the fourth quintile.

**TABLE 3:** The association between sleep hours/work hours ratio (quintiles) and the risk of new-onset diabetes (China, 2022).

Sleep time/work time (everyday)	Model 1		Model 2		Model 3	
	HR (95% CI)	*p* value	HR (95% CI)	*p* value	HR (95% CI)	*p* value
Q1	Ref		Ref		Ref	
Q2	0.76 (0.53–1.10)	0.145	0.80 (0.55–1.15)	0.220	0.82 (0.57–1.19)	0.303
Q3	0.98 (0.66–1.44)	0.901	1.03 (0.70–1.53)	0.867	1.05 (0.69–1.59)	0.828
Q4	0.53 (0.35–0.80)	0.003	0.55 (0.36–0.85)	0.007	0.57 (0.36–0.91)	0.018
Q5	0.51 (0.33–0.77)	0.002	0.49 (0.31–0.76)	0.002	0.66 (0.42–1.06)	0.083

Model 2: adjusted for age at baseline (<45, 45-<60, ≥60), sex (male/female), residence (urban or rural) and education (Illiteracy, Primary school, middle school, high school or above).

Model 3 was further adjusted for BMI (<24 kg/m^2^, ≥24 kg/m^2^), SBP (<140 mmHg, ≥140 mmHg), DBP (<90 mmHg, ≥90 mmHg), smoking status (yes/no), alcohol consumption (yes/no), drinking tea (yes/no), drinking coffee (yes/no), total energy intake (continuous), total fat intake (continuous), total carbohydrate intake (continuous) and total protein intake (continuous).

Abbreviations: BMI, body mass index; SBP, systolic blood pressure; DBP, diastolic blood pressure; HR, hazard ratio; CI, confidence interval.

**FIGURE 3:**
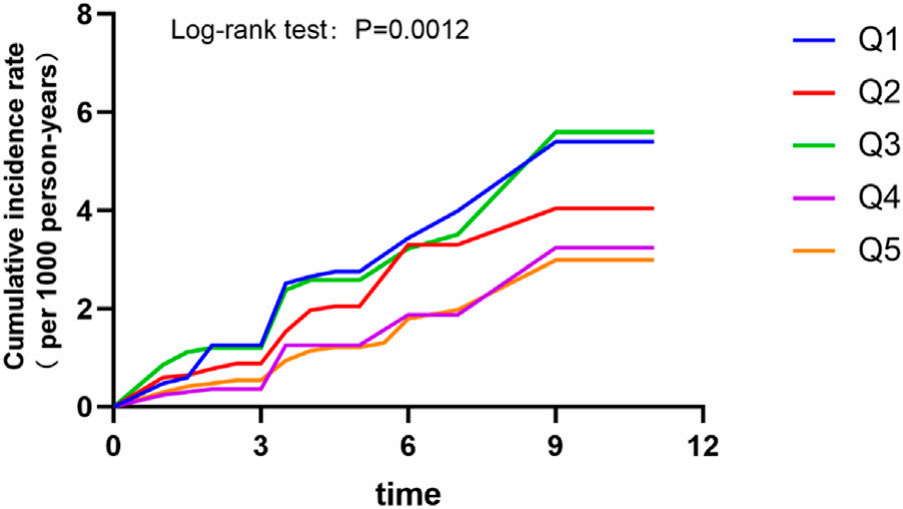
Cumulative incidence rates by quintiles of sleep hours/work hours ratio (China, 2022).

Meanwhile, Kaplan-Meier analysis was utilized to explore the effect of SH/WH ratio on the cumulative probability of diabetes (Figure 3). It was observed that those in the fourth quintile had the lower cumulative incidence of diabetes.

Sensitivity analyses were performed to test the robustness of the results. First, we reanalyzed age, BMI, systolic and diastolic blood pressure as continuous variables, and the results did not change significantly (Supplementary Table S1). Second, when missing values for BMI (500), SBP (437), DBP (437), smoking status (10), alcohol consumption (74), drinking tea (11), drinking coffee (12) were imputed with the use of multiple imputation, we concluded the same results (Supplementary Table S2). Finally, there was no substantial change when we excluded participants whose diabetes event occurred in the first 2 years of follow-up (Supplementary Table S3).

## Discussion

This study is the first relatively large retrospective cohort study to examine the joint effect of sleep and work duration on new-onset diabetes in Chinese adults. We expressed the pooled effect as the ratio of daily sleep duration to daily work hours and found a non-linear L-shaped association between the ratio and the risk of diabetes. The results showed that people who slept more than their working hours each day had a 25% lower risk of developing diabetes than those who slept less than their working hours after adjusting for potential variables. When SH/WH ratio was assessed as quintiles, compared to participants in the first quintile, a significantly lower risk (HR: 0.57, 95% CI: 0.36–0.91) of diabetes was found in those in the fourth quintile (1.10–<1.37).

Diabetes and its complications are important causes of the burden of death and disability worldwide. The increasing incidence of diabetes has stimulated the search for new etiological factors. Although there is a strong genetic basis for individual susceptibility to diabetes, current epidemiologic evidence suggests that diabetes can be prevented through lifestyle changes [16, 17]. In recent years, sleep duration has been considered to be independently associated with the occurrence and development of diabetes. Some studies have shown that both short and long sleep duration are associated with an increased risk of diabetes [6, 8, 18, 19]. However, a new study found that accelerometer-measured short but not long sleep duration was associated with a higher risk of incident diabetes [20]. What is now recognized by most people is that short sleep increases the incidence of diabetes through a variety of mechanisms [21–24]. However, the relationship and underlying mechanisms regarding prolonged sleep and diabetes are not clear. According to a consensus statement from the American Academy of Sleep Medicine and Sleep Research Society, adults should sleep 7 or more hours per night on a regular basis to promote optimal health while it is not sure whether sleeping more than 9 h is associated with health risk [25]. It is worth noting that there is no official and clearly defined optimal daily sleep time for individuals. There is less research on the relationship between work hours and diabetes, but previous evidence suggests that longer work hours increase the risk of the disease [10, 12]. Our study provided a new idea for assessing the joint effect of sleep and work duration on new-onset diabetes in the general population. In our study, we found that daily sleep, including daytime and nighttime hours, should exceed the working hours in order to effectively prevent the progression of diabetes. The optimal SH/WH ratio was 1.10–<1.37. In other words, if an adult works 8 h a day, he needs to get a total of 8.80–<10.96 h of sleep per day to reduce the risk of developing diabetes. Due to the fast pace and high pressure of modern society, adults tend to shorten their sleep time to work. Our study shows that adults who sleep less than their working hours each day are at high risk of developing diabetes. This may serve as a cautionary tale for Chinese adults.

To our knowledge, this is the first large national population-based sample study to investigate the combined effect of sleep and work duration on diabetes and to determine the optimal ratio. There were still some limitations to this study. Firstly, as a retrospective study, we cannot ignore the effect of other unknown confounders on our results although we adjusted for multiple covariates. In addition, since this study was conducted in a Chinese population, it may not be applicable to non-Chinese populations. In the present study, the information about new-onset diabetes, sleep duration and work hours were self-reported by the participants, which may be subject to reporting and measurement bias. Further large prospective cohort studies are necessary in the future to elucidate the correlation between combined effect of sleep and work hours and diabetic events in the Chinese population.

### Conclusion

In conclusion, there was a L-shaped association between SH/WH ratio and the risk of diabetes. After adjustment for covariates, individuals with ratios between 1.10 and 1.37 had the lowest risk for incident diabetes. Our study suggests that balancing work and sleep schedules is important to prevent the onset of diabetes.

## Data Availability

The data and study materials that support the findings of this study can be found from the CHNS official website (http://www.cpc.unc.edu/projects/china).
