# Supplementary material for: Sleeping More Hours Per Day Than Working Can Prevent New-Onset Diabetes
Source: Int J Public Health. 2023 Dec 8;68:1606634. doi: 10.3389/ijph.2023.1606634 (PMC10739384; doi:10.3389/ijph.2023.1606634)
Supplement: Supplementary file 1 [file Table1.DOCX]

Supplementary Table 1

| sleep time /work time  (everyday) | Model 1 | | Model 2 | | Model 3 | |
| --- | --- | --- | --- | --- | --- | --- |
|  | HR (95% CI) | P | HR (95% CI) | P | HR (95% CI) | P |
| Q1 | Ref |  | Ref |  | Ref |  |
| Q2 | 0.76(0.53-1.10) | 0.145 | 0.80(0.56-1.15) | 0.235 | 0.84(0.58-1.21) | 0.348 |
| Q3 | 0.98(0.66-1.44) | 0.901 | 1.03(0.70-1.52) | 0.890 | 0.97(0.64-1.48) | 0.896 |
| Q4 | 0.53(0.35-0.80) | 0.003 | 0.55(0.36-0.84) | 0.007 | 0.58(0.37-0.92) | 0.020 |
| Q5 | 0.51(0.33-0.77) | 0.002 | 0.47(0.30-0.73) | 0.001 | 0.70(0.44-1.11) | 0.127 |

Abbreviations: BMI, body mass index;SBP, systolic blood pressure;DBP, diastolic blood pressure； HR, hazard ratio; CI,confidence interval

Model 2: adjusted for age at baseline (continuous), sex (male/female), residence (urban or rural) and education (Illiteracy, Primary school,middle school , high school or above).

Model 3: was further adjusted for BMI (continuous), SBP (continuous), DBP (continuous), smoking status(yes/no), alcohol consumption(yes/no), drinking tea(yes/no), drinking coffee(yes/no), total energy intake (continuous), total fat intake (continuous), total carbohydrate intake (continuous) and total protenion intake (continuous).

Supplementary Table 2

| sleep time /work time  (everyday) | Model 1 | | Model 2 | | Model 3 | |
| --- | --- | --- | --- | --- | --- | --- |
|  | Coefficient  (95% CI) | P | Coefficient  (95% CI) | P | Coefficient  (95% CI) | P |
| Q1 | Ref |  | Ref |  | Ref |  |
| Q2 | -0.27(-0.63,0.09) | 0.145 | -0.23(-0.59,0.14) | 0.220 | -0.17(-0.54,0.20) | 0.369 |
| Q3 | -0.02(-0.41,0.36) | 0.901 | -0.03(-0.36,0.42) | 0.867 | 0.22(-0.39,0.44) | 0.918 |
| Q4 | -0.64(-1.06,-0.22) | 0.003 | -0.59(-1.02,-0.16) | 0.007 | -0.46(-0.90,-0.11) | 0.044 |
| Q5 | -0.68(-1.10,-0.26) | 0.002 | -0.72(-1.16-0.27) | 0.002 | -0.38(-0.84,0.87) | 0.1112 |

Missing values for BMI, SBP, DBP, smoking status, alcohol consumption, drinking tea, drinking coffee were imputed with the use of multiple imputation.

Model 2: adjusted for age at baseline (<45, 45-<60, ≥60), sex (male/female), residence (urban or rural) and education (Illiteracy, Primary school, middle school, high school or above).

Model 3 was further adjusted for BMI (<24kg/m2, ≥24kg/m2), SBP (<140mmHg, ≥140mmHg), DBP (<90mmHg, ≥90mmHg), smoking status (yes/no), alcohol consumption(yes/no), drinking tea(yes/no), drinking coffee(yes/no), total energy intake (continuous), total fat intake (continuous), total carbohydrate intake (continuous) and total protein intake (continuous).

Abbreviations: BMI, body mass index; SBP, systolic blood pressure; DBP, diastolic blood pressure; HR, hazard ratio; CI, confidence interval

Supplementary Table 3

| sleep time /work time  (everyday) | Model 1 | | Model 2 | | Model 3 | |
| --- | --- | --- | --- | --- | --- | --- |
|  | HR (95% CI) | P | HR (95% CI) | P | HR (95% CI) | P |
| Q1 | Ref |  | Ref |  | Ref |  |
| Q2 | 0.77(0.54-1.10) | 0.152 | 0.80(0.55-1.14) | 0.215 | 0.83(0.57-1.20) | 0.312 |
| Q3 | 0.97(0.66-1.43) | 0.889 | 1.03(0.70-1.51) | 0.895 | 1.05(0.59-1.60) | 0.966 |
| Q4 | 0.53(0.35-0.80) | 0.003 | 0.55(0.36-0.84) | 0.006 | 0.58(0.36-0.91) | 0.018 |
| Q5 | 0.50(0.33-0.77) | 0.001 | 0.48(0.31-0.76) | 0.002 | 0.66(0.42-1.05) | 0.082 |

Excluded participants whose diabetes event occurred in the first 2 years (n=7348)

Model 2: adjusted for age at baseline (<45, 45-<60, ≥60), sex (male/female), residence (urban or rural) and education (Illiteracy, Primary school, middle school, high school or above).

Model 3 was further adjusted for BMI (<24kg/m2, ≥24kg/m2), SBP (<140mmHg, ≥140mmHg), DBP (<90mmHg, ≥90mmHg), smoking status (yes/no), alcohol consumption(yes/no), drinking tea(yes/no), drinking coffee(yes/no), total energy intake (continuous), total fat intake (continuous), total carbohydrate intake (continuous) and total protein intake (continuous).

Abbreviations: BMI, body mass index; SBP, systolic blood pressure; DBP, diastolic blood pressure; HR, hazard ratio; CI, confidence interval
